# RETRACTION: Effect of Cefuroxime Intracameral Injection Antibiotic Prophylactic on Postoperative Endophthalmitis Wound Post‐Cataract: A Meta‐Analysis

**DOI:** 10.1111/iwj.70504

**Published:** 2025-04-18

## Abstract

**RETRACTION:** WangM. , LiuY. , and DongH. , “Effect of Cefuroxime Intracameral Injection Antibiotic Prophylactic on Postoperative Endophthalmitis Wound Post‐Cataract: A Meta‐Analysis,” International Wound Journal 20, no. 5 (2023): 1376–1383, 10.1111/iwj.13984.36346142 PMC10088833

The above article, published online on 08 November 2022, in Wiley Online Library (http://onlinelibrary.wiley.com/), has been retracted by agreement between the journal Editor in Chief, Professor Keith Harding; and John Wiley & Sons Ltd. Following an investigation by the publisher, all parties have concluded that this article was accepted solely on the basis of a compromised peer review process. The editors have therefore decided to retract the article. The authors did not respond to our notice regarding the retraction.

**RETRACTION:** M. Wang , Y. Liu , and H. Dong , “Effect of Cefuroxime Intracameral Injection Antibiotic Prophylactic on Postoperative Endophthalmitis Wound Post‐Cataract: A Meta‐Analysis,” International Wound Journal 20, no. 5 (2023): 1376–1383, 10.1111/iwj.13984.36346142 PMC10088833

The above article, published online on 08 November 2022, in Wiley Online Library (http://onlinelibrary.wiley.com/), has been retracted by agreement between the journal Editor in Chief, Professor Keith Harding; and John Wiley & Sons Ltd. Following an investigation by the publisher, all parties have concluded that this article was accepted solely on the basis of a compromised peer review process. The editors have therefore decided to retract the article. The authors did not respond to our notice regarding the retraction.

